# Clinical Significance of Combined Weight‐Bearing and Non‐Weight‐Bearing Positions and MRI Examination in Evaluating Genu Varus

**DOI:** 10.1111/os.12766

**Published:** 2020-10-04

**Authors:** Shan Zhu, Zhi Wang, Feng He

**Affiliations:** ^1^ Department of Radiology Tianjin University Tianjin Hospital Tianjin China; ^2^ Department of Biomedical Engineering, School of Precision Instrument and Optoelectronic Engineering Tianjin University Tianjin China

**Keywords:** False negative, Genu varus, Non‐weight‐bearing position, Osteoarthritis

## Abstract

**Objective:**

To siscuss the clinical significance of the early diagnosis of knee varus and knee osteoarthritis with the combination of negative position and non‐negative position and radiography.

**Methods:**

One hundred and eighty patients whose femorotibial angles <182° (genu varus positive) measured by X‐ray at the weight‐bearing position and femorotibial angles ≥182° (genu varus negative) measured by X‐ray at the non‐weight‐bearing position were selected as the sample group from those patients who received knee joint Magnetic resonance imaging (MRI) examination from July 2015 to July 2017. One hundred and eighty patients whose femorotibial angles ≥182° (genu varus negative) measured at both the weight‐bearing position and the non‐weight‐bearing position were selected as the control group. Femorotibial angles of both groups were respectively measured, to respectively compare and analyze the effect of non‐weight‐bearing false‐negative genu varus on the occurrence and severity of injury of medial meniscus and femorotibial articular cartilage. The two groups of patients had no previous history of knee surgery, and no lower limb fracture, inflammation, tumor, metabolic bone disease, or congenital disease.

**Results:**

The weight‐bearing tibiofemoral angles of the non‐weight‐bearing false‐negative genu varus group and the negative genu varus group (180.998° ± 0.589°) were lower than the non‐weight‐bearing tibiofemoral angles (182.501° ± 0.290°), and they were positively correlated (*t* = −15.048, *P* < 0.01). The non‐weight‐bearing knee varus medial meniscus incidence of false‐ negative group. Medial meniscus injury that occurred in the sample group were 86.7% (156/180) in the anterior horn, 91.7% (165/180) in the body, 88.3% (159/180) in the posterior horn. Medial meniscus injury that occurred in the control group were 46.7% (84 /180) in the anterior horn, 40.6.3% (73/180) in the body, 43.3% (78/180) in the posterior horn. The incidence of degenerative groups, the differences were statistically significant. The incidence and severity of injury were as follows: medial meniscus anterior horn (*χ* ^*2*^ = 41.966, *P* = 0.000), body (*χ* ^*2*^ = 104.94, *P* = 0.000), posterior horn (*χ* ^*2*^ = 81.025, *P* = 0.000). The incidence and severity of medial meniscus injury in the non‐weight‐bearing knee varus false negative group was higher than in the control group. The non‐weight‐bearing knee varus false‐negative group medial tibiofemoral articular cartilage degeneration rate was 95.0% (171/180); in the control group, medial tibiofemoral articular cartilage degeneration was 65.1% (117/180). Two medial tibiofemoral articular cartilage degeneration incidence were statistically significant. The incidence and severity of injury were as follows: medial tibiofemoral articular cartilage (*χ* ^*2*^ = 50.625, *P* = 0.000). The incidence and severity of medial tibiofemoral articular cartilage injury in the non‐weight‐bearing knee varus false negative group was higher than in the control group.

**Conclusion:**

The combined weight‐bearing position and non‐weight‐bearing position imaging examination for diagnosing the non‐weight‐bearing false‐negative genu varus patients at an early date is of significant importance to the early diagnosis and treatment of knee osteoarthritis.

## Graphical abstract

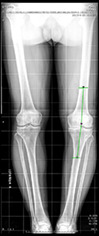

## Introduction

As the population's life span is gradually prolonged, the incidence of osteoarthritis gradually increases. Among the population aged above 60 years, 50% have the symptoms of osteoarthritis, and partial ones have imaging changes. Among the population aged above 75 years, 80% have the symptoms of osteoarthritis, and almost all have the imaging changes^1^.

Osteoarthritis often attacks the knee joint, which conforms to the epidemiological pattern of osteoarthritis. Knee osteoarthritis is caused by many factors, and the knee osteoarthritis and genu varus are of reciprocal causation^2^. Genu varus can lead to changes in the mechanical distribution of the inside and outside of the knee joint. As a result, the articular cartilage on one side of the knee joint is subjected to excessive pressure, which leads to gradual wear and degeneration of the joint. Continuous degenerative changes in the joints will lead to joint instability, collateral ligament injury and relaxation, which will aggravate the degree of knee varus and further aggravate knee osteoarthritis.

The measurement of lower limb line can be divided into weight‐bearing and non‐weight‐bearing position. In the early stage, the lower limb strength line was measured in the non‐weight‐bearing position or supine position. Soott first recognized the importance of weight‐bearing position X‐rays in evaluating joint strength lines after knee and hip replacements in 1985. Most physicians believe that the measured results of degenerative osteoarthropathy（OA） patients at the weight‐bearing position of the lower extremities can more truly reflect the correlation between the alignment of the lower extremities under the pathological state of the patients. When there are bone defects and ligament laxity, the results of non‐weight‐bearing position measurement may underestimate the degree of lower extremity deformity. At the same time, when the X‐ray of the whole lower limb was used to measure the lower limb strength line at the weight‐bearing position, the variation between observers was only 1.3°, which was of high reliability. Therefore, most physicians recommend the use of weight‐bearing position to measure the lower limb strength line. However, it is difficult to popularize the full length X‐ray of both lower limbs measurement because of its expensive equipment and high technical requirements.

The genu varus is evaluated by measuring the lower limb alignment tibiofemoral angle. The lower limb alignment is measured at the weight‐bearing position and the non‐weight‐bearing position. The overseas scholars believe that measurement in weight‐bearing lower limbs can more accurately reflect the correlation of lower limb alignment arrangement under pathological status; thus, the weight‐bearing full length X‐ray of both lower limbs is preferred^3^, ^4^. Meanwhile, they believe that when the lower limb alignment is measured with weight‐bearing full length X‐ray of both lower limbs, the inter‐observer variability is less and reliability is higher^5^.

It is generally believed that the best method to measure the lower limb alignment is to draw the anatomical axis and the mechanical axis on the weight‐bearing full length X‐ray of both lower limbs and then measure the parameters of each alignment. However, when taking full‐length images of the lower limbs, due to the lack of standard shooting position, there are problems such as artificial parallax limb and femur rotation, knee flexion, and femur prosthesis occlusion, which will affect the accuracy of measurement of the lower limb alignment. In addition, since there is no unified determination method for anatomical axis and mechanical axis, the accuracy of measurement will also be affected.

The weight‐bearing full length X‐ray of both lower limbs requires more on the equipment and technical conditions, thus, some doctors prefer the non‐weight‐bearing measurement^6^, ^7^. In this study, the tibiofemoral angle was measured respectively by the lower limb weight‐bearing full‐length radiography and non‐weight‐bearing knee joint orthotopic radiography to evaluate the genu varus. Thus, the effect of the non‐weight‐bearing false‐negative genu varus on occurrence and development of knee osteoarthritis was understood: to perform early diagnosis on the patients with non‐weight‐bearing false‐negative genu varus, take the preventive treatment and relieve the development of osteoarthritis. However, due to the high requirements of equipment and technical conditions for the full‐length X‐ray measurement of the lower limbs in the weight‐bearing position, some doctors prefer to use the non‐weight‐bearing position measurement.

1. In this study, we measured the tibiofemoral angle of both the full‐length X‐ray photograph of the lower extremity in the weight‐bearing position and positive X‐ray photograph of the knee joint in the non‐weight‐bearing position for the same patient, and evaluated genu varus by combining the tibiofemoral angle measured in the two positions.
2. By comparing the two measurement methods in this study, we can understand the influence of the occurrence of genu varus in non‐weight‐bearing position on the occurrence and development of osteoarthritis, so as to prove the important clinical significance of full‐length X‐ray photography of lower limbs in weight‐bearing position.
3. In this study, the degree of meniscus and articular cartilage degeneration of the subjects was understood in combination with the knee joint MRI scan evaluation, so as to make early diagnosis of non‐weight‐bearing false negative genu varus patients and take preventive treatment to alleviate the development of osteoarthritis.

## Patients and Methods

### *Patient Demographics*

Each patient was provided informed consent for participation in the study. This retrospective study was conducted in accordance with the Declaration of Helsinki (Ethical Principles for Medical Research Involving Human Subjects) and was approved by the Ethics Committee of Tianjin University Tianjin Hospital.

Inclusion criteria: i) patients who received knee joint MRI examination from July 2015 to July 2017 in our hospital were examined with full‐length radiography of both lower limbs in the erection position and knee joint orthotopic radiography in the supine position; ii) 180 patients, whose tibiofemoral angle ≥182° measured at the non‐weight‐bearing position and besides <182° measured at weight‐bearing position were selected as the non‐weight‐bearing false‐negative genu varus group; iii) 180 patients whose tibiofemoral angle ≥182° measured at both the non‐weight bearing position and the weight‐bearing position were selected as the negative genu varus group; iv) MRI scans of the knee joints in the non‐weight‐bearing false‐negative inversion group, and evaluating the degree of degeneration of articular cartilage, meniscus, etc. compared with the negative inversion group; v) a retrospective study.

Patients who received knee joint MR examination from July 2015 to July 2017 in our hospital were examined with full‐length radiography of both lower limbs in the erection position and knee joint orthotopic radiography in the supine position, to respectively measure the weight‐bearing and non‐weight‐bearing tibiofemoral angle angle of knee joint at the affected side.

180 patients, whose tibiofemoral angle ≥182° measured at the non‐weight‐bearing position and besides <182° measured at weight‐bearing position were selected as the non‐weight‐bearing false‐negative genu varus group. 180 patients whose tibiofemoral angle ≥182° measured at both the non‐weight bearing position and the weight‐bearing position were selected as the negative genu varus group. The sample group included 96 males and 84 females, aged 40–70 years; 157 cases had left knee symptoms, and 23 cases had right knee symptoms. The control group included 65 males and 115 females, aged 40–70 years; 126 cases had left knee symptoms, and 54 cases had right knee symptoms. Patients in both groups received no knee joint surgery and had no fracture of lower limb, inflammation, tumor, metabolic bone disease, or congenital diseases.

### *Scanning Technology*

MRI examination was performed on a 3‐T MRI scanner (GE750, GE Healthcare, Waukesha, WI), using a dedicated 8‐channel knee coil (Invivo Inc., Gainesville, FL). The patients were in the supine position with the knee extended. The sequences obtained during MRI imaging were Sagittal 3D T1 weighed SPGR (TR/ TE 6.3 ms/3.1 ms), Sagittal FS PDWI (TR/TE 2228 ms/22.0 ms), Coronal FS PDWI (TR/TE 1846 ms/26.5 ms), and Traverse STIR T2WI (TR/TE 4000 ms/100.6 ms) at 1‐mm slice thickness and increment, respectively. Respective fields of view were adjusted to patient anatomy and size.

DR manufactured by GE is equipped with a bracket holding 41 cm × 41 cm cassettes, and a 41 cm × 128 cm locating plate, and a 41 cm × 128 cm anti‐scatter grid. Patients receiving DR examination were in the standing position^8^, with the film distance of 1.8 m, and the exposure condition of 70–75 kV, gamma ray exposure of 20–25 mas. The knee joint horizontal position was taken as the center, and the film was exposed in one time. The obtained figures and data were processed with the computer full‐leg software system into the full‐length image of lower limbs. The patients were in the supine position, with the knee joint at the affected side straightened, the kneecap facing the front, the film distance of 1 m, and the exposure condition of 50 MV.

### *Research Indicators and Imaging Evaluation*

#### *Measurement of the Femorotibial Angle of the Knee Joint*

This study is based on the measurement of tibiofemoral angle on the X‐ray of the knee joint in the Brouwer position.

**Fig. 1:**
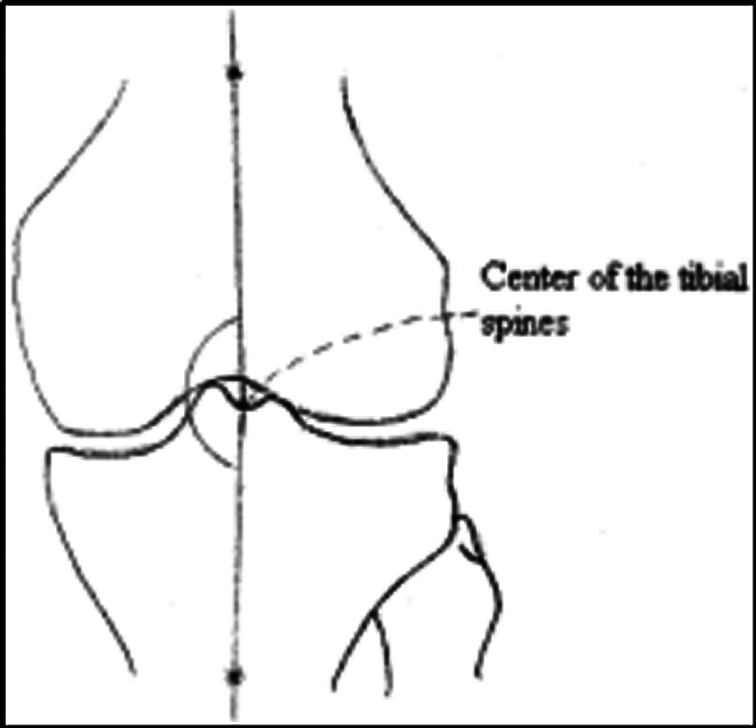
Measurement of Tibiofemoral Angle.

The longitudinal axis of the femur center refers to the line from the femoral shaft center and the midpoint of the medial and lateral femoral shaft at 10 cm above the knee joint plane. The longitudinal axis of the tibia center refers to the line from the tibial shaft center to the midpoint of the medial and lateral tibial shaft at 10 cm above the knee joint plane^9^. The inner intersected angle means the tibiofemoral angle (Fig. 1).

**Fig. 2:**
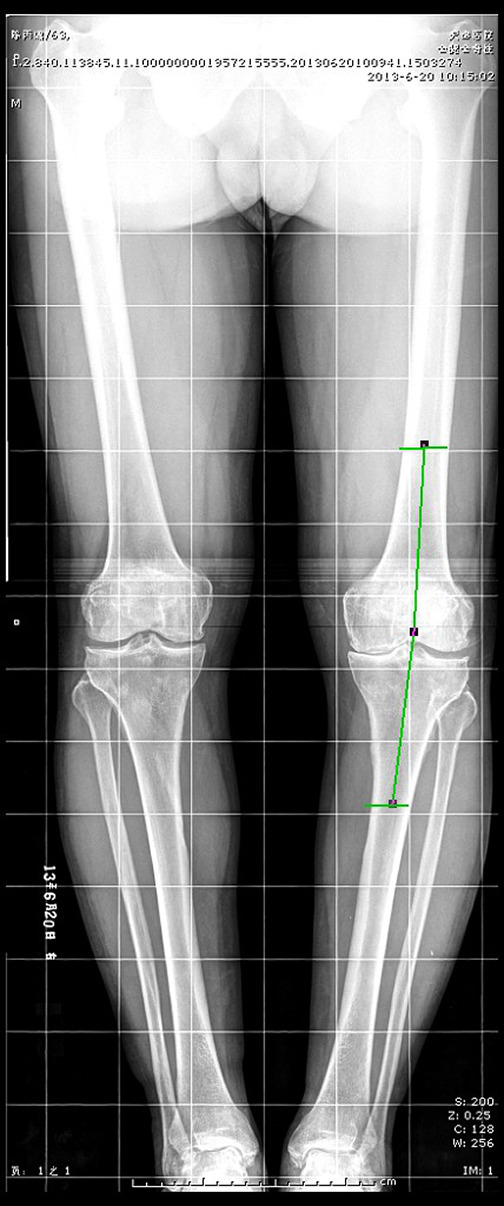
Full‐length Radiography of Both Lower Limbs at Weight‐bearing Position.

**Fig. 3:**
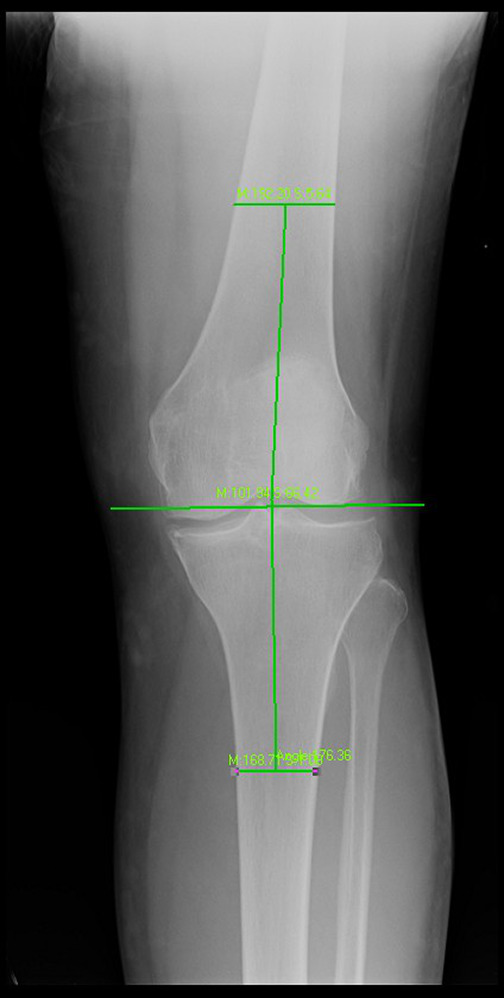
Affected knee joint orthotopic radiography at non‐weight‐bearing position (supine position).

Here, the inner included tibiofemoral angle was measured respectively at the weight‐bearing position (Fig. 2) and the non‐weight‐bearing position (Fig. 3). Brouwer *et al*.^10^ believed that the medial tibiofemoral angle <182° indicated the genu varus, 182°–184° indicated normal, while >184° indicated genu valgum. In this paper, the medial tibiofemoral angle <182° was the standard for diagnosing the positive genu varus.

### *Diagnosis Standards for Injury of Medial Femorotibial Articular Cartilage*

ICRS grading of articular cartilage injury was used.

Grade 0: normal articular cartilage is recorded as grade 0, the cartilage surface is smooth, and there is no mixed signal on each layer of MRI.

Grade I: the surface of cartilage is not smooth, the signal layer of cartilage parenchyma is blurred, there is low signal performance on T1WI, high signal performance on STIR, the thickness of cartilage damage is less than 50%, and there are patchy abnormal signals in subchondral bone.

Grade II: the surface of the joint is irregular, and the T1WI and T2WI signals of the cartilage surface are jagged, and the deep endplate of the cartilage begins to appear fuzzy, and the shape also becomes irregular. The signal level of the cartilage and subchondral bone is blurred, and the T1WI is low signal performance, STIR is high signal, in addition, there is hyperplasia or sclerosis of subchondral bone.

Grade III: there is severe irregularity in articular cartilage, cartilage thinning, severe deficiency, blurred subchondral endplate, obvious bone hyperplasia or sclerosis, T1WI shows low signal, STIR shows high signal.

Grade IV: full thickness of cartilage is lost, subchondral bone hyperplasia and sclerosis are serious, and the subchondral endplate is exposed, T1WI is low signal performance, STIR is high signal performance.

**Fig. 4:**
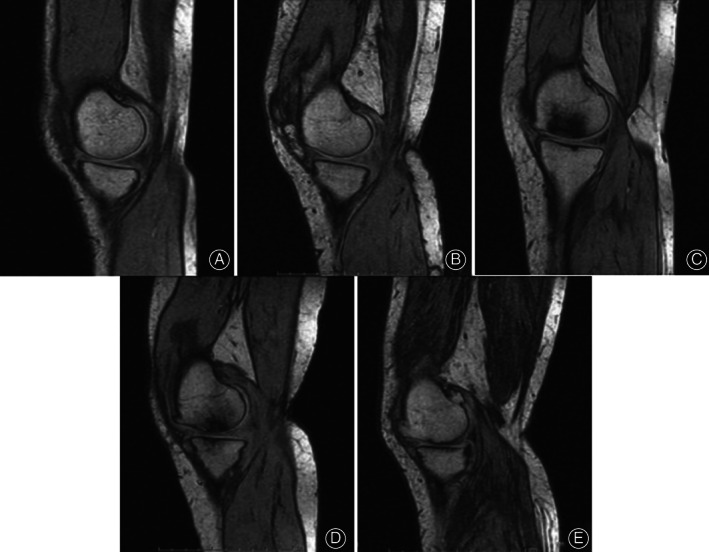
MRI T1WI for Grading of Knee‐joint Cartilage Injury. Grade 0: Normal, MRI indicates the joint cartilage signal was even, with normal thickness and smooth surface (A). Grade I: Joint cartilage was thickened, the signal for partial cartilage was decreased, the joint cartilage surface was smooth (B). Grade II: Local joint cartilage was irregular and coarse, and cartilage thickness was not apparently reduced (C). Grade III: The joint cartilage was irregularly thinned (D). Grade IV: Local joint cartilage has a defect, and partial subchondral bone was exposed (E).

Refer to MRI grading standards of knee‐joint cartilage injury^11^, the injury severity of medial femorotibial articular cartilage was from mild to severe (1 to 5), as shown in Fig. 4.

#### *Diagnosis Standards for Injury of Anterior Horn, Body, and Posterior Horn of Medial Meniscus*

Meniscus of knee joint can be divided into grade 0–III on MRI，It is clinically recognized that there are different MRI manifestations between levels.

0: Normal signal on MRI.

I: The fat and PDWI sequences show internal points flaky or circular‐like hypersensitivity in the meniscus shadow, not reaching the edge of the meniscus.

II: In the fat presaturated PDWI sequence, high signals in horizontal or diagonal stripes appear inside the meniscus, not reaching the edge of the meniscus.

III: The fatty presaturated PDWI sequence shows high signal in the meniscus bar, reaching the edge of the meniscus.

**Fig. 5:**
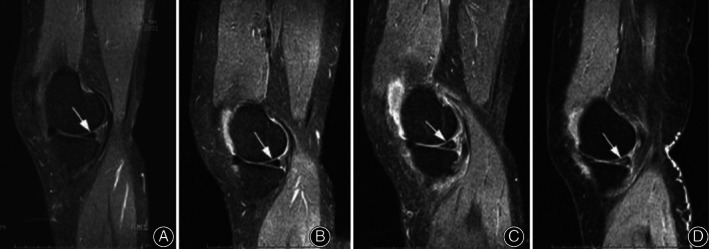
T2WI STIR for grading of meniscus injury. (A) Grade 0: Normal, MRI indicated the medial meniscus was integral, in smooth margin and in even and uniformed low signal (as shown by the arrow). (B) Grade I: The isolated dotted high signal indicated the meniscus articular surface margin was not invaded, medial meniscus was integral and in smooth margin (as shown by the arrow). (C) Grade II: The linear high signal indicated the meniscus articular surface margin was not invaded (as shown by the arrow). (D) Grade III: The high signal of medial meniscus indicated articular surface margin was invaded, accompanied with irregular meniscus (as shown by the arrow).

Stoller^12^ put forward the meniscus injury MRI grading standards, in which the medial meniscus injury was graded from mild to severe (1 to 4)^13^, as shown in Fig. 5.

### *Statistical Analysis*

Statistical analysis was performed with SPSS 20.0 software. The weight‐bearing and non‐weight‐bearing femorotibial angles were expressed in mean ± SD, in‐group paired *t* test and Pearson correlation analysis were used to compare the in‐group mean. The injury severity of anterior horn, body, and posterior horn of medial meniscus, and that of medial femorotibial articular cartilage in the non‐weight‐bearing false‐negative genu varus group and the control group were compared by the Mann–Whitney *U* test. And the injury rate was compared by the χ^2^ test. *P* < 0.05 indicated the difference was statistically significant.

## Results

Difference of sex (*χ* ^*2*^ = 14.903, *P* = 0.426), age (*t* = 12.556, *P* = 0.359), and the affected side (*χ^2^* = 1.170, *P* = 0.279) of two groups patients was not statistically significant.

### *Comparison of the Femorotibial Angle of Knee Joint at Weight‐Bearing and Non‐Weight‐Bearing Positions*

For 120 cases in the non‐weight‐bearing false‐negative genu varus group and negative genu varus group, the difference of femorotibial angles at weight‐bearing and non‐weight‐bearing positions were compared, finding the femorotibial angle at the weight‐bearing position was less than that at the non‐weight‐bearing position, difference was statistically significant (*t* = −15.048, *P* < 0.01) (Table 1).

**TABLE 1:** Comparison of Femorotibial Angles at the Weight‐bearing Position and the Non‐weight‐bearing Position (°)

Groups	Weight‐bearing position	Non‐weight‐bearing position	*t* value	*P* value
Sample group (*n* = 180)	180.998 ± 0.589	182.501 ± 0.290	−31.84	0.000
Control group (*n* = 180)	182.485 ± 0.284	183.478 ± 0.285	−33.624	0.000

sample group: non‐weight‐bearing false‐negative genu varus group; control group: negative genu varus group.

### *Relationship between Non‐Weight‐Bearing False‐Negative Genu Varus and Medial Meniscus Degeneration of the Knee Joint*

MRI results showed that injury of medial meniscus in the non‐weight‐bearing false‐negative genu varus group: anterior horn 83.3% (150/180), body 91.7% (165/180), and posterior horn 88.3% (159/180). Injury of medial meniscus in the control group: anterior horn 52.0% (104/180), body 40.6% (73/180), and posterior horn 43.3% (78/180).

Comparison about degeneration of medial meniscus anterior horn (*χ* ^*2*^ = 41.966, *P* = 0.000), body (*χ* ^*2*^ = 104.94, *P* = 0.000), and posterior horn (*χ* ^*2*^ = 81.025, *P* = 0.000) between the non‐weight‐bearing false‐negative genu varus group and the control group showed the difference was statistically significant (Table 2).

**TABLE 2:** Injury Severity of Medial Meniscus Anterior Horn, Body and Posterior Horn between the Non‐weight‐bearing False‐negative Genu Varus Group and the Control Group (Cases)

Groups	Cases	Degeneration of meniscus anterior horn				Degeneration of meniscus body				Degeneration of meniscus posterior horn			
		Grade 0	Grade I	Grade II	Grade III	Grade 0	Grade I	Grade II	Grade III	Grade 0	Grade I	Grade II	Grade III
sample group	180	30	20	104	32	15	12	99	54	21	51	69	39
Control group	180‐	96	54	24	6	107	51	16	6	102	36	33	9
*Z* value		−10.036				−12.878				−8.968			
*P* value	‐	0.000				0.000				0.000			

sample group: non‐weight‐bearing false‐negative genu varus group; control group: negative genu varus group.

Results found that the injury rate and severity of medial meniscus in the non‐weight‐bearing false‐negative genu varus group was apparently higher than those in the control group (*P* = 0.000), indicating the patients in the former group were easier to be attacked by the injury and degeneration of medial meniscus of the knee joint.

### *Relationship Between Non‐Weight‐Bearing False‐Negative Genu Varus and Degeneration of the Medial Femorotibial Articular Cartilage*

MRI results found that the degeneration of the medial femorotibial articular cartilage in the non‐weight‐bearing false‐negative genu varus group was 95.0% (171/180), and that in the control group was 65.0% (117/180). The comparison between both groups showed statistical significance (*χ* ^*2*^ = 50.625, *P* = 0.000) (Table 3).

**TABLE 3:** Comparison of Injury Severity of Medial Femorotibial Articular Cartilage Between the Non‐weight‐bearing False‐negative Genu Varus Group and the negative genu varus Group (Cases)

Groups	Cases	Injury and degeneration of medial femorotibial articular cartilage				
		Grade 0	Grade I	Grade II	Grade III	Grade IV
Sample group	180	9	15	36	69	51
Control group	180	63	51	48	12	6

sample group: non‐weight‐bearing false‐negative genu varus group; control group: negative genu varus group.

The degeneration severity and incidence of medial femorotibial articular cartilage in the non‐weight‐bearing false‐negative genu varus group were apparently higher than those in the negative genu varus group (*Z* = ‐11.383, *P* = 0.000), indicating that the patients in non‐weight‐bearing false‐negative genu varus group were easier to be attacked by the injury and degeneration of medial femorotibial articular cartilage.

## Discussion

Pauwels^14^ put forward the concept of anatomical axis and mechanical axis in 1980 about X‐ray measurement of lower limb alignment. Owing to the different anatomical axis and mechanical axis of tibia and femur, the tibia mechanical axis angle was also different from its anatomical axis angle. Thus, when discussing the genu varus, either the mechanical axis or the anatomical axis should be specified for measurement. In this study, the non‐weight‐bearing knee joint orthotopic X‐ray image could not completely cover the femoral head and full‐length femur, thus, the femur mechanical axis could not be determined. In order to guarantee the consistency of femorotibial angle measurement at the weight‐bearing position and the non‐weight‐bearing position^15^, ^16^, in this study, the inner angle of anatomical axes of tibia and femur was used to evaluate the occurrence of genu varus.

### *Relationship between Femorotibial Angles of Knee Joints Measured by X‐ray at the Weight‐Bearing Position and the Non‐Weight‐Bearing Position*

Knee osteoarthritis and genu varus are of reciprocal causation: genu varus can change the distribution of inner and outside loads of knee joint, to lead to the degenerative changes in the joint. The persistent degenerative change of the joint can gradually make the knee joint unstable, thus, the genu varus will be aggravated^2^. Thus, early discovery of genu varus in clinic is of great significance to prevention and early treatment of knee osteoarthritis.

This study found that for the sample group and the control group, the mean femorotibial angle measured at the weight‐bearing position was 180.998° ± 0.589°, and that measured at the non‐weight‐bearing position was 182.501° ± 0.290°. That measured at the weight‐bearing position was less than that measured at the non‐weight‐bearing position and they were positively correlated. This study believed that the genu varus at the weight‐bearing position was more apparent than that at the non‐weight‐bearing position, namely, the measurement of lower limb alignment at the weight‐bearing position was more sensitive in early diagnosis of genu varus, and conducive to its early observation.

### *Effect of Non‐Weight‐Bearing False‐Negative Genu Varus on the Medial Meniscus Degeneration*

MRI results found that the degeneration of the medial tibiofemoral articular cartilage in the non‐weight‐bearing false‐negative genu varus group was 95.0% (171/180), and that in the control group was 65.0% (117/180).

And the meniscus injury rate and severity in the non‐weight‐bearing false‐negative genu varus group were apparently higher than those in the control group, indicating that the non‐weight‐bearing false‐negative genu varus could apparently enhance the occurrence of meniscus injury.

Medial meniscus can hold good coupling of the medial tibiofemedial joint and joint stability, conduct load, absorb vibration, lubricate the joint, and provide body feelings. Such functions are dependent on the complete adhesion of medial meniscus with medial tibial plateau and the hoop stress formed by the annular fibers of medial meniscus^17^. When the knee joint is in weight‐bearing status, the meniscus medial meniscus will bear and conduct most of the stress loads. This study believed that when the non‐weight‐bearing false‐negative genu varus occurred, the knee joint center in the weight‐bearing status would be biased toward the inner rather than the normal knee joint; thus, the adduction torque of the medial femorotibial joint would increase, clearance of the medial tibiofemoral joint would be narrowed, and as a result, the medial meniscus would be extruded and worn. Even if the non‐weight‐bearing position can make the adduction torque of the medial tibiofemoral joint recover and knee joint gravity return, the frequently abnormal change of gravity makes the fibers in medial meniscus repeatedly stretch out; thus, a series of injuries of medial meniscus will occur.

### *Effect of Non‐Weight‐Bearing False‐Negative Genu Varus on the Degeneration of the Femorotibial Articular Cartilage*

This study showed that when the non‐weight‐bearing false‐negative genu varus occurred, the degeneration rate (97%) and severity of tibiofemoral articular cartilage were higher than the rate (62%) and severity in the control group.

When the knee joint is in the weight‐bearing status, the medial meniscus conducts most of the stress loads, and other loads will be conducted by the direct contact of articular cartilage of medial femoral condyle and tibial medial plateau. This study believed that when the non‐weight‐bearing genu varus occurred, as the adduction torque of medial tibiofemoral joint changed abnormally, the medial femorotibial joint pressure at the weight‐bearing position would be apparently higher than the normal knee joint. Even if the adduction torque of medial femorotibial joint can be recovered and medial femorotibial joint pressure can be recovered, the frequent change of pressure between femorotibial articular cartilage due to change of position, mutual extrusion, and friction of femorotibial articular cartilage will result in frequent changes of subchondral bone stress, thus, the articular cartilage degeneration, subchondral marrow edema, and osteophyte will occur^18^, ^19^.

### *Conclusions and Clinical Significance of this Study*

This study found that the measurement of lower limb alignment by X‐ray at the weight‐bearing position was more sensitive in early diagnosis of genu varus, and conducive to its early observation. The degeneration rate and severity of tibiofemoral articular cartilage and medial meniscus for the non‐weight‐bearing false‐negative genu varus patients are apparently higher than those for negative genu varus patients^20^. Thus, it is necessary to take the combined weight‐bearing imaging examination and non‐weight‐bearing imaging examination to diagnose the non‐weight‐bearing false‐negative genu varus patients, positively find out the reason for weight‐bearing genu varus, correct the irregular arrangement of lower limb alignment with rehabilitation training or orthopedic surgery, and perform the osteoporosis therapy. It is of great significance clinically in preventing or postponing the occurrence and development of knee osteoarthritis.
